# A Review of Clinical Practice Guidelines and Treatment Recommendations for Cancer Care in the COVID-19 Pandemic

**DOI:** 10.3390/cancers12092452

**Published:** 2020-08-29

**Authors:** Alberto Zaniboni, Michele Ghidini, Francesco Grossi, Alice Indini, Francesca Trevisan, Alessandro Iaculli, Lorenzo Dottorini, Giovanna Moleri, Alessandro Russo, Ivano Vavassori, Alessandra Brevi, Emanuele Rausa, Luigi Boni, Daniele Dondossola, Nicola Valeri, Antonio Ghidini, Gianluca Tomasello, Fausto Petrelli

**Affiliations:** 1 Oncology Unit, Fondazione Poliambulanza, 25124 Brescia, Italy; azaniboni@alice.it; 2 Oncology Unit, Fondazione IRCCS–Ca’ Granda, Ospedale Maggiore Policlinico Milan, 20122 Milan, Italy; mghido@hotmail.it (M.G.); fg1965@libero.it (F.G.); alice.indini@gmail.com (A.I.); gianluca.tomasello@gmail.com (G.T.); 3 Radiotherapy Unit, ASST Bergamo Ovest, 24047 Treviglio (BG), Italy; trevisan.francesca1@gmail.com; 4 Oncology Unit, ASST Bergamo Est, 24068 Seriate (BG), Italy; aleiaculli@gmail.com (A.I.); ldottorini@gmail.com (L.D.); 5 Centro Servizi, Direzione Socio-Sanitaria, ASST Bergamo Ovest, 24047 Treviglio (BG), Italy; giovanna.moleri@libero.it; 6 Surgical Oncology Unit, ASST Bergamo Ovest, 24047 Treviglio (BG), Italy; alessandrorusso70@yahoo.it; 7 Urology Unit, ASST Bergamo Ovest, 24047 Treviglio (BG), Italy; ivano.vavassori0@alice.it; 8 Otorhinolaryngology-Head and Neck Surgery Unit, ASST Bergamo Ovest, 24047 Treviglio (BG), Italy; brevialessandra@gmail.com; 9 General Surgery 1 Unit, ASST Papa Giovanni XXIII, 24127 Bergamo, Italy; emarausa@yahoo.it; 10 Department of Surgery, Fondazione IRCCS–Ca’ Granda, Ospedale Maggiore Policlinico, University of Milan, 20122 Milan, Italy; luigi.boni@policlinico.mi.it; 11 General and Liver Transplant Surgery Unit, Fondazione IRCCS–Ca’ Granda, Ospedale Maggiore Policlinico Milan, 20122 Milan, Italy; daniele.dondossola@policlinico.mi.it; 12 Department of Pathophysiology and Transplantation, Università degli Studi of Milan, 20122 Milan, Italy; 13 Division of Molecular Pathology and Centre for Evolution and Cancer, The Institute of Cancer Research, London SW7 3RP, UK; nicola.valeri@icr.ac.uk; 14 Department of Medicine, The Royal Marsden Hospital, London SW3 6JJ, UK; 15 Oncology Unit, Casa di cura Igea, 20129 Milan, Italy; antonioghidini@hotmail.com; 16 Oncology Unit, Medical Sciences Department, ASST Bergamo Ovest, 24047 Treviglio (BG), Italy

**Keywords:** cancer, patients, treatment, COVID-19, pandemic

## Abstract

The COVID-19 pandemic has inevitably caused those involved in cancer care to change clinical practice in order to minimize the risk of infection while maintaining cancer treatment as a priority. General advice during the pandemic suggests that most patients continue with ongoing therapies or planned surgeries, while follow-up visits may instead be delayed until the resolution of the outbreak. We conducted a literature search using PubMed to identify articles published in English language that reported on care recommendations for cancer patients during the COVID-19 pandemic from its inception up to 1st June 2020, using the terms “(cancer or tumor) AND (COVID 19)”. Articles were selected for relevance and split into five categories: (1) personal recommendations of single or multiple authors, (2) recommendations of single authoritative centers, (3) recommendations of panels of experts or of multiple regional comprehensive centers, (4) recommendations of multicenter cooperative groups, (5) official guidelines or recommendations of health authorities. Of the 97 included studies, 10 were personal recommendations of single or multiple independent authors, 16 were practice recommendations of single authoritative cancer centers, 35 were recommendations provided by panel of experts or of multiple regional comprehensive centers, 19 were cooperative group position papers, and finally, 17 were official guidelines statements. The COVID-19 pandemic is a global emergency, and has rapidly modified our clinical practice. Delaying unnecessary treatment, minimizing toxicity, and identifying care priorities for surgery, radiotherapy, and systemic therapies must be viewed as basic priorities in the COVID-19 era.

## 1. Introduction

Since the first report and identification of the responsible agent, the disease associated with the novel beta-coronavirus SARS-CoV2 (COVID-19) has spread globally, with an estimated 3.5 million cases and more than 20,000 deaths by end of April 2020. The explosion has been overwhelming, disrupting almost every healthcare system of involved countries and finding unprepared even those funded by robust economic resources. Healthcare professionals have suddenly seen the dawn of a completely new disease. COVID-19 has promptly been understood to be a “systemic disease” rather than a mere interstitial pneumonia. 

Managing such a new clinical condition involves the challenge of dealing with both a lack of evidence and a lack of experience. However, unlike the previous HIV pandemic in the 80s, for the first time in the modern age we have had to face the problems of high volumes and an unprecedented rapidity of spread. Professionals from all specialties have suddenly found themselves being forced to become respiratory physicians, infectious disease specialists, and anesthetists; in this framework, the lack of knowledge in biology, epidemiology, pathophysiology, immune response, and treatment has highlighted the unmet need for uniformity and systematic review of current evidence. 

Many national and international oncologic scientific societies have developed indications and guidelines for oncologists to follow in daily clinical practice. The aim of this review is to collect and discuss the current available guidelines and clinical practice recommendations for oncologists so far, as these professionals are faced with the challenge of continuing to deliver optimal care to cancer patients during the COVID-19 pandemic.

## 2. Results

### 2.1. Personal Recommendations or Single Authoritative Center Statements

Several local, national, and international recommendations for the management of cancer patients have emerged during the COVID-19 pandemic [1,2,3,4,5,6,7,8,9,10,11,12,13,14,15,16]. For example, there have been suggestions that surveillance should be delivered remotely for patients who have completed cancer treatment. In other cases, treatment might be deferred or completely avoided if the impact on quality of life is thought to be marginal. Only when treatment has a potentially curative role should it not be delayed. Examples are chemotherapy responsive tumors such as testicular, ovarian, and small cell lung cancers [4]. Moreover, a switch from intravenous to oral correspondent formulations (e.g., etoposide and vinorelbine) may be a valid indication [5]. Less intensive strategies are important especially in the cases of older and vulnerable patients. For example, in metastatic breast cancer patients, maintenance endocrine therapies after completing chemotherapy might represent a sound option in the elderly population. In general, older patients with cancer should not be systematically excluded from cancer treatments during COVID-19. However, it is worth noticing that, in case of COVID-19 infection and related complications during anticancer treatments, elderly patients are less likely to experience benefit from intensive unit admission and need for invasive mechanical ventilation highlighting the need for detailed upfront discussions about ceiling of care among oncologists, patients, and their families in this scenarios.

Not only medical treatment but also surgical indications for cancer patients have been influenced by the COVID-19 pandemic. The Massachusetts General Hospital has proposed a multidisciplinary approach for triage of resectable patients. Using a virtual conference modality, the team identified five different profiles of patients suitable for oncologic surgery in a 7–10 day time frame. In particular, patients in the window of resectability after preoperative chemotherapy and cancer types with aggressive behaviors (e.g., triple negative breast cancer) are prioritized. In addition, diagnostic surgeries, second parts of staged procedures (after completion of the first part), and interventions due to onset of acute symptoms (e.g., gastrointestinal bleeding) are considered urgent and non-delayable [6]. In colorectal cancer surgery, a minimally invasive approach was suggested, with the prioritization of cancer-related emergencies (to be treated within 2 weeks). Conversely, a deferral period of up to 2 months was proposed in the case of surgeries for curable tumors. In early-stage disease, surgery could be deferred even later than 2 months from diagnosis [7]. Similarly, surgery for early-stage lung cancer was promoted both in stages I and IIa disease, the former with a low risk of progression and of COVID-19 infection, the latter with a high risk of progression and a low risk of infection. However, for stage IIb disease (low risk of progression but high risk of COVID-19 infection), conservative management with a follow-up up to 3 months before potential surgery is advisable. Finally, stage III disease, with a high risk of progression and of COVID-19 infection, requires specific medical treatments [8]. Among non-surgical therapies in lung cancer, adjuvant chemotherapy after surgery may be delayed up to 4 months after surgery without affecting patient survival. Chemotherapy with adjuvant and maintenance intent may be postponed or switched to oral formulation, while oral targeted drugs for patients with sensitive gene mutations should be administered without combination chemotherapy in order to avoid adverse events. As far as immunotherapy is concerned, treatment with checkpoint inhibitors has low immunosuppressive potential and avoiding it during a coronavirus infection may unfairly deprive these patients of an active class of drugs. However, special consideration should be given to patients suffering from immune-related adverse events because of their prolonged exposition to immunosuppressive agents, such as steroids [9]. On the whole, immunotherapy may be suspended or postponed in the case of stable disease and, generally, there is no need to administer it regularly during the epidemic period [10]. In non-small cell lung cancer, neoadjuvant chemotherapy for locally advanced resectable disease and sequential or concurrent chemoradiotherapy for stage III disease should be started when possible. In the advanced stages, first-line treatment and palliative or ablative radiotherapy outside the lung should not be delayed, either. Similarly, in small-cell lung cancer, both concurrent chemoradiotherapy and first-line therapy are both indicated with palliative or curative purposes [11].

In addition to medical and surgical treatment, radiation treatment should also be omitted or shortened in times of COVID-19 infection. Breast cancer experts from the Memorial Sloan Kettering Cancer Center in New York suggested the omission of radiotherapy in the case of ductal carcinoma in situ, in patients aged 70 and older, and in the case of invasive estrogen-receptor positive disease smaller than 3 cm in size without nodal involvement and with negative resection margins. However, in the cases of ductal carcinoma in situ with lesions bigger than 2.5 cm, inadequate resection margins, or high-grade disease and in invasive estrogen-receptor positive tumors in younger patients, experts recommended a delay in treatment of 8–12 weeks after surgery. In general, hypofractionated or accelerated breast radiotherapy regimens are preferred in order to reduce treatment duration. High priority indications for breast radiotherapy are the diagnosis of inflammatory breast cancer and residual node positivity after neoadjuvant treatment, the presence of node-positive (N2) disease, recurrent disease, a diagnosis of triple negative node-positive disease, and extensive lymphovascular invasion [12].

In a pandemic phase with reduced availability of intensive/subintensive care beds, treatment strategies may prioritize medical treatment aimed at downstaging the disease until the peak of the pandemic has disappeared and the number of intensive care unit beds has increased. This approach is recommended in the treatment of ovarian cancer, where first-step surgery is preferred, especially in the case of otherwise healthy patients. In these unprecedented times, indications may be inverted and neoadjuvant chemotherapy could become the standard of care [13].

Management of cancers of the head and neck during a COVID-19 infection is an important matter to discuss because of the multidisciplinary features of management of these cancers. Moreover, patients with tracheostomy or total laryngectomy have a high risk of virus aerosolization and require special attention in terms of strategies to minimize the risks of infection [14]. Treatment of low-risk tumors like differentiated thyroid cancer should be delayed, with minimally invasive and transoral surgical approaches preferred over open and major surgery [15]. In the case of concomitant chemoradiotherapy indication for locally advanced disease, medical treatment should be omitted for patients who have comorbidities or who are older than 70. Similarly, sequential treatment with ciplatin-based induction chemotherapy should not be administered for these patients. Exclusive and definitive radiotherapy should be limited to simultaneous integrated boost techniques in the standard or accelerated schedule, in order to reduce treatment duration to 1 week, shorter than the sequential technique. In the case of salivary gland tumors, it is indicated to delay post-operative radiotherapy up to 12 weeks after surgery [15].

### 2.2. Recommendations of Panels of Experts or Regional Cooperative Centers

Different groups of experts tried to provide recommendations at a regional or more general level. For example, by describing the approach used to manage patients with cancer during a large-scale, respiratory syndrome-coronavirus hospital outbreak in Saudi Arabia in 2015, the authors offered a plan to help manage oncology services to prevent harm to patients or staff [16]. The plan focused on managing oncology services, infected patients, preventing any new infections in patients or staff, ensuring the continuity of cancer care, and incorporating measures to sustain these interventions far into the postoutbreak period.

Similarly, authors from Iran provided recommendations in order to limit the exposure of cancer patients to medical environments and to modify the treatment modalities in a manner that reduces the probability of myelosuppression. Such recommendations include delaying elective diagnostic and therapeutic services, shortening the treatment course, or prolonging the interval between treatment courses [17]. Specific precautions to prevent virus spread among cancer patients and cancer care providers were also suggested by Indian authors who additionally provided a table of myths and misinformation about COVID-19. This Table 1, based on advice published by the WHO, proved useful in mitigating panic in cancer patients [18].

Among the areas of China hardest hit by COVID-19 was Heilongjiang province. A series of protocols were established when the first confirmed case emerged, and authors summarized their experience in medical management strategies including protection of medical staff, reallocation of medical resources, plans for hierarchical treatment, and utilization of a network platform [19]. 

In an attempt to help cancer centers in low-resource settings, authors from Colombia created some adjusted recommendations such as (1) assuring social containment; (2) moving tumor boards and scientific meetings to virtual modalities; (3) changing of immunotherapy to 4 or 6 week schedules for selected patients, switching to oral therapies for advanced cases with intravenous treatments, and temporarily discontinuing noncritical therapies, such as bisphosphonates or denosumab; (4) using strict selection criteria for in-hospital chemotherapy. According to these authors, only potentially curative chemotherapy with severe toxicity profile should be delivered to inpatients for acute leukemias, high-grade lymphomas or soft tissue sarcomas [20].

Simple and straightforward guidance on decisions about immediate cancer treatment involving different treatment modalities (i.e., surgery, chemotherapy, and radiotherapy) during the COVID-19 crisis was also generated by Kutikov and colleagues from the Fox Chase Cancer Center [21]. Based on the risk for significant morbidity from COVID-19 (comorbidities need to be considered) and on the risk of cancer progression in case of treatment delay, patients were prioritized in disease groups to streamline clinical decisions and avoid deferral of treatment in specific high-risk groups.

Another panel of experts from the US reviewed strategies for mitigating the transmission of COVID-19 in an effort to reduce morbidity and mortality of cancer patients and healthcare workers [22].

Outside China, Italy had one of the largest COVID-19 outbreaks. Lambertini and colleagues offered practical and interesting suggestions on how to implement cancer care during the COVID-19 outbreak [23]. Their approach was summarized by the acronym YOP, which outlines priorities to protect: (1) Yourself (physicians) and their families, both at work and in their personal life, by following all official instructions, respecting lifestyle restrictions, and focusing on proper use and adequate stocks of personal protective equipment (PPE); (2) Oncological care of patients, by deferring what can be delayed but trying, as much as possible, to minimize the impact of the pandemic on the usual standard of care; (3) Patients themselves from being infected, by making any possible effort to minimize the risks and giving continuous direction and appropriate official information.

A number of experts tried to provide specific recommendations based on tumor subgroups. For example, leaders from the Magee Breast Cancer Program (from Surgery, Medical Oncology, Radiation Oncology, Plastic Surgery, Pathology, and Genetics) came to a consensus and prepared a statement that may guide breast care professionals in diagnosis, treatment, and follow-up during the COVID-19 pandemic [24]. Similarly, a panel of breast surgeons from Turkey highlighted the national and international approach to the crisis, and wrote a document to be used in routine clinical practice which may provide beneficial recommendations for breast surgery in the state of emergency [25]. Breast Journal panelists proposed how to triage, prioritize, and organize breast cancer cases during a COVID-19 outbreak [26]. Marijnen et al. provided recommendations for rectal cancer treatment using ESMO guidelines as a platform [27]. They encourage modulating treatments (from TME surgery alone to short course radiotherapy (RT) + neoadjuvant chemotherapy (CT) or CTRT for more advanced cases) and depict scenarios of various risk groups. 

A further panel of experts provided suggestions and recommendations for the management of urological conditions during COVID-19 crisis in Brazil and other low- and middle-income countries. Specifically, the panel reached a consensus to prepare a practical guide for urologists based on the recommendations from the main Urologic Associations, as well from as data from the literature supporting the suggested management [28]. Additional recommendations on how to reorganize routine urological practice and prioritize systemic therapies for genitourinary malignancies came from Italy and USA, respectively [29,30]. Interestingly, the Editorial Team of the International Journal of Gynecological Cancer took the initiative to use established guidelines to prepare a practical tool in order to be able to propose strategies to optimize care of gynecological oncology patients [30].

Recommendations on dermatologic surgery during the COVID-19 pandemic were also published by experts from the UK who clearly stated that elective surgery such as the excision of benign lesions and cosmetic procedures should be postponed [31]. Conversely, patients with locally aggressive tumors (e.g., melanoma, dermatofibroma sarcoma protuberans, Merkel cell carcinoma, microcystic adnexal carcinoma) should proceed as soon as possible [31].

Finally, Italian radiation therapists provided recommendations on how to safely run a radiation oncology department and listed practical recommendations for radiation therapy during the COVID-19 outbreak, based on specific cancer care contexts [32,33]. Furthermore, a RADS framework (Remote visits, Avoidance, Deferment, and Shortening of radiotherapy) was created by an international panel of experts and applied to determine the appropriate management for prostate cancer during the global COVID-19 pandemic. Consensus was reached that all aspects of patient visits, treatment, and overall resource utilization can be reduced for all identified stages of prostate cancer treated with radiotherapy [34].

### 2.3. Recommendations of Multicenter Cooperative Groups

Al-Shamsi et al., on behalf of the International Collaborative Group, outlined various aspects of cancer care for patients being treated during the pandemic in a paper published in The Oncologist [35]. They discussed economic issues, allocation of resources, treatment of outpatients and hospitalized cancer patients, risk of infecting patients, and surgical considerations. In this exhaustive review, the authors addressed some of the current challenges associated with the managing of cancer patients during the COVID-19 pandemic and provided topical recommendations. In particular, lung cancer, hematopoietic stem cell transplantation, psychological aspects, and clinical research were discussed. Thureau et al., on behalf of the GEMO group (a European study group for bone metastases), discussed the topic of palliative RT for symptomatic bone metastases [36]. They indicated a single 8 Gy fraction as the recommended schedule for the palliation of bone pain. For spinal cord compression, surgical treatment should be prioritized whenever possible for all patients with a life expectancy of more than a few months. In cases where surgery is not indicated, exclusive RT may be indicated with a similar fractionation used for treating bone pain. Penel et al., on behalf of the French Sarcoma Group, briefly identified the major topics of sarcoma treatment [37]. In suspected COVID-19 cases, primary treatment should be postponed for at least 15 days after the symptoms start. Otherwise, all other treatment settings in COVID-19 negative patients should reflect the current practice. 

The Consensus Statement from Thoracic Surgery Outcomes Research Network determined the ideal priorities for thoracic surgery in cancer patients [38]. They outlined situations that need immediate surgery (for staging, for symptomatic or node positive cancers, or after neoadjuvant therapy), delayed surgery (up to 3 months, for isolated lung nodules, thymomas or indolent histologies, for example), or alternative treatment modalities (as stereotactic body RT). When almost all hospital centers are dedicated to COVID-19, all cases except for those with perforated cancer of esophagus, septic patients, or patients with surgical complications may be reasonably delayed until after the pandemic has resolved. 

Finally, the gynecological FRANCOGYN group discussed the topic of gynecological cancers during the pandemic [39]. They prefer neoadjuvant chemotherapy in stage III ovarian cancer with cytoreduction (without HIPEC) performed after six cycles. Cervical cancer can be managed with definitive CTRT to avoid surgical burden and low-risk endometrial cancers can be resected even after a 1–2 month waiting period. 

### 2.4. Official Guidelines or Recommendations of Health Authorities

Several papers providing regional or international guidelines were published in these weeks [40,41,42,43,44,45,46,47,48,49,50]. Three were national guidelines (n = 2 French and n = 1 Lebanese) and eight came from international societies. Four were RT guidelines (for lymphomas, head and neck, lung, and breast cancers), four were specific surgical guidelines (n = 1 gynecological, n = 1 urological, n = 2 head and neck malignancies), one discussed infection prevention, and one was the guidelines of the Society of Surgical Oncology (SSO). Finally, a European hematologist discussed prevention and treatment of cancer patients at risk of with COVID-19 infection.

The ILROG consensus was published by the International Society for Radiotherapy treatment in Lymphomas. They advised three possible strategies for RT delivery during the pandemic: omitting, delaying, and shortening the RT course. In particular, they consider omitting RT in the case of a palliative setting, localized low-grade lymphomas if completely excised, localized nodular lymphocyte-predominant Hodgkin lymphoma if wholly excised, and for consolidation RT for diffuse large B cell lymphomas/aggressive non Hodgkin lymphomas in patients who have completed the full CT course and achieved a complete remission. 

Bartlett et al., on behalf of the SSO, briefly outlined the surgical indications for significant cancer types according to stage. Except for lung and gynecological cancers that were not part of these guidelines, they described surgical indications of the main cancer subtypes (breast, thyroid, abdominal, melanoma, and sarcoma). They endorse neoadjuvant therapies in many cases (breast, gastroesophageal, pancreatic, peritoneal, and high-grade sarcomas) with the deferral of resection procedures in many low-risk settings. Coles et al. reported on international guidelines for breast cancer RT. They reported five statements/recommendations regarding low-risk breast cancer, fractionation, elderly with ER+ breast cancer, boost necessity, and nodal RT. 

A group of French authors published local recommendations for the protection of cancer patients from COVID-19 infection. They suggest minimizing hospital visits, using telemedicine and phone calls to replace safety visits, replacing intravenous drugs with oral drugs, and adjusting the dosage of CT and RT to reduce the frequency of hospital admissions. They list three treatment settings with reduced priority: (1) the curative setting, (2) the palliative (first line) setting for younger and fit patients or patients with at least 5 years of life expectancy, and (3) the palliative therapy setting in other cases. 

ASTRO, ESTRO, and select Asia-Pacific countries provided head and neck RT guidelines through a modified rapid Delphi process. They reported agreement in many domains such as priority areas, treatment dose adjustment, RT delay, indications for surgery, and management of outpatients. Finally, Fakhry et al., on behalf of French societies for head and neck cancers, discussed surgical priorities in these patients. Group A refers to life-threatening emergencies (shortness of breath, hemorrhage) where immediate treatment is required, Group B refers to cancers for whom postponing the treatment beyond 1 month could have a negative prognostic impact for the patient and where management should not be delayed, and Group C refers to cancers for which treatment can be postponed for at least 6–8 weeks without any significant prognostic impact.

## 3. Materials and Methods 

We conducted a literature search using PubMed to identify articles published in English language that reported on cancer patient care recommendations during the COVID-19 pandemic from inception up to 1st June 2020, using the terms “(cancer or tumor) AND (COVID-19)” (Table 1) [1,2,3,4,5,6,7,8,9,10,11,12,13,14,15,16,17,18,19,20,21,22,23,24,25,26,27,28,29,30,31,32,33,34,35,36,37,38,39,40,41,42,43,44,45,46,47,48,49,50,51,52,53,54,55,56,57,58,59,60,61,62,63,64,65,66,67,68,69,70,71,72,73,74,75,76,77,78,79,80,81,82,83,84,85,86,87,88,89,90,91,92,93,94,95,96,97].

**Figure 1:**
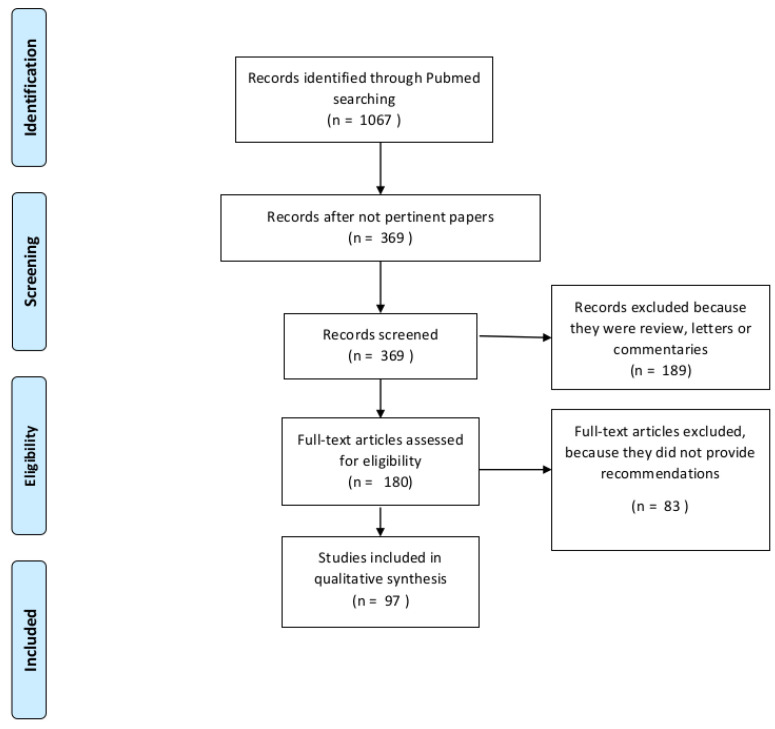
Flow diagram of included studies.

Of the 97 included studies, 10 were personal recommendations of single or multiple independent authors, 16 were practice recommendations of single authoritative cancer centers, 35 were recommendations provided by panels of experts or by multiple regional cooperative centers, 19 were cooperative group position papers, and finally, 17 were official guidelines statements. The flow diagram of the included studies is reported in Figure 1.

## 4. Conclusions

We systematically searched and collected all recommendations produced for cancer care during the COVID-19 pandemic era at various levels (personal view, single institution position, panel of experts, cooperative groups, and specific guidelines). Several aspects of treatment were discussed by the authors (surgery, CT, RT, supportive therapies) and these recommendations may judiciously guide care of patients in oncology setting during this worldwide emergency situation. The COVID-19 pandemic is a global emergency, and this has rapidly modified our clinical practice. Delaying of unnecessary treatment, minimizing the burden of toxicity, and identifying care priorities for surgery, radiotherapy, and systemic therapies settings must be viewed as basic priorities in the COVID-19 era and may shape cancer care services in the future. 

Clinicians are aware about the various recommendations that are being provided for care of cancer from a local to an international point of view. International guidelines are probably less suitable for universal (worldwide) use. In fact, there are enormous differences between various countries and continents due to economic resources available, to the different evolution of the pandemic, to the presence or not of local (hub) high volume centers for the treatment of oncological pathologies, etc. In conclusion, we believe that oncologists, surgeons, and radiation oncologists should refer to the indications of their proper, national, scientific societies. The rapid evolution of epidemiology of pandemic, however, makes a continuous update of clinical practice guidelines, a necessity.

## Figures and Tables

**Table 1:** Characteristics of included studies.

Author/Year	Journal	Country	Type of Study	Disease	Synthesis of Main Recommendations
Ansarin/2020	Acta Otorhinolaryngol Ital	Italy	Personal view or multi-authors review	H&N	Recommendations about surgery and compromise between the necessary cancer treatments and the risk of infection
Banna/2020	ESMO Open	Italy and Switzerland	Personal view or multi-authors review	Lung	A decisional tool to support oncologists and physicians in treatment for patients with lung cancer: primum non nocere
Cafarotti/2020	J Thorac Oncol	Switzerland	Personal view or multi-authors review	Lung	An algorithm of care to balance the risk of dying from cancer or from potentially fatal infection
Di Saverio/2020	Colorectal Dis	Italy	Personal view or multi-authors review	Colorectal	Management of patients needing surgery to mitigate some risks and reduce exposure to other patients
Falandry/2020	J Geriatr Oncol	France	Personal view or multi-authors review	Various	Challenges with the management of older patients with cancer
Kattan/2020	Immunotherapy	France and Lebanon	Personal view or multi-authors review	Various	A careful selection of the most efficacious anti-tumor weaponry with the lower risk of weaning the patients’ immune system
Mandato/2020	Obstet Gynecol	Italy	Personal view or multi-authors review	Ovarian	Finding new effective strategies in cancer care is mandatory (allocate resources and real-life treatment)
Schrag/2020	JAMA	US	Personal view or multi-authors review	Various	Planning for resuming cancer treatment and screening to mitigate harms; changes will transform cancer treatment
Scotté/2020	Eur J Cancer	France	Personal view or multi-authors review	Various	Use of telemedicine for monitoring and optimizing referral of Covid-19-positive patients with cancer (CAPRI programme)
Zhao/2020	Thorac Cancer	China	Personal view or multi-authors review	Lung	Recommendations and suggestions of individualized treatment strategies and management of common adverse events for patients with lung cancer
Braunstein/2020	Adv Radiat Oncol	US	Single authoritative center view	Breast	The parsimonious application of breast radiotherapy without compromising long term oncologic outcomes
Davis/2020	Immunotherapy	Australia	Single authoritative center view	Immunotherapy	Suggestions about immunotherapy use during pandemic
De Felice/2020	Radiother Oncol	Italy	Single authoritative center view	H&N	To offer adequate individualized treatment recommendations based on both the epidemic situation and the patient’s own condition
Gentileschi/2020	Eur J Surg Oncol	Italy	Single authoritative center view	Skin	Skin cancer management
Kligerman/2020	Head Neck	US and Hong Kong	Single authoritative center view	H&N	To help minimize the risk of aerosolization and SARS-CoV-2 exposures in head and neck cancer patients with tracheostomy and TL
Qadan/2020	Ann Surg	US	Single authoritative center view	Various	A multidisciplinary team approach for triage of elective cancer surgery
Salari/2020	Oral Oncol	Iran	Single authoritative center view	H&N	The role of virtual multidisciplinary team meetings
Tagliaferri/2020	J Eur Acad Dermatol Venereol	Italy	Single authoritative center view	Skin	Management of skin cancers during COVID-19 era
Tasoulis/2020	Eur J Surg Oncol	UK	Single authoritative center view	Breast	Position of The Royal Marsden regarding breast cancer surgery
Wang/2020	JAMA Oncol	China	Single authoritative center view	Various	More attention should be paid to patients with cancer as a special population
Thompson/2020	Ann Surg	USA	Single authoritative center view	Breast	Revised indication of neoadjuvant endocrine therapy for the treatment of early stage estrogen receptor positive breast cancer
Li/2020	Leukemia	China	Single authoritative center view	Chronic myeloid leukemia	Questionnaires of subjects with chronic myeloid leukemia during COVID-19 pandemic
Sharma/2020	Liver Int	UK	Single authoritative center view	Hepatocellular Cancer (HCC)	Recommendations for the treatment of HCC during COVID-19 pandemic
Yerramilli/2020	Adv Radiat Oncol	USA	Single authoritative center view	Various	Use of hypofractionated radiation therapy for patients requiring palliation for oncologic emergencies
Valenza/2020	Tumori	Italy	Single authoritative center view	Various	Screening of patients accessing to a Comprehensive Cancer Center with real-time PCR of nose-throat swabs
Viale/2020	Oncologist	Italy	Single authoritative center view	Breast	Personalized strategies for optimal breast cancer management
Cakmak/2020	Eur J Breast Health	Turkey	Panel of experts or regional recommendations	Breast	Recommendations about timing of surgery of breast cancer according to biology and risk
Carneiro/2020	Int Braz J Urol	Brazil	Panel of experts or regional recommendations	Urologic	Suggestions and recommendations for the management of urological conditions in times of COVID-19 crisis in Brazil and other low- and middle-income countries
Cinar/2020	J Natl Compr Canc Netw	US	Panel of experts or regional recommendations	Various	Strategies to mitigate transmission of COVID-19 in an effort to reduce morbidity and mortality associated with the disease for patients with cancer and for the healthcare workers
Curigliano/2020	Breast	International	Panel of experts or regional recommendations	Breast	Advise on how to triage, prioritize, and organize diagnostic procedures, surgical, radiation, and medical treatments in breast cancer
Dietz/2020	Breast Cancer Res Treat	US	Panel of experts or regional recommendations	Breast	Recommendations for prioritization, treatment and triage of breast cancer patients during the COVID-19 pandemic
Ficarra/2020	Minerva Urol Nefrol	Italy	Panel of experts or regional recommendations	Urologic	Strategies for the reorganization of urological routine practice and a set of recommendations to facilitate the process of rescheduling surgical activity
Finley/2020	Can J Surg	Canada	Panel of experts or regional recommendations	Various	Recommendations about cancer surgery by Canadian surgeons
Head and Neck Surgery Treatment Guidelines Consortium/2020	Head Neck	US	Panel of experts or regional recommendations	H&N	Head and Neck cancer treatment according to site and stage of disease is presented
Jazieh/2020	JCO Glob Oncol	Saudi Arabia	Panel of experts or regional recommendations	Various	It is important to have a robust mechanism to prioritize patients to ensure the provision of timely care while preventing further harm by guiding staff to provide care in safety
Koffman/2020	Am J Hematol	US	Panel of experts or regional recommendations	CLL	Recommendation about CLL treatment by a CLL panel of experts
Kowalski/2020	Head Neck	International	Panel of experts or regional recommendations	H&N	Procedures essential to maintain safety of otolaryngologists and maxillofacial surgeons exposed to the greatest risk of infection
Krengli/2020	Adv Radiat Oncol	Italy	Panel of experts or regional recommendations	Various	To adopt preventive measures and recommendations for patients, professionals, and clinical operations to minimize the risk of infection while safely treating cancer patients
Kutikov/2020	Ann Intern Med	US	Panel of experts or regional recommendations	Various	Risks must be balanced carefully, public health strategies implemented thoroughly, and resources utilized wisely
Lalani/2020	Can Urol Assoc J	Canada	Panel of experts or regional recommendations	Genitourinary	Recommendations to assist in prioritizing systemic therapies for patients with genitourinary cancers
Lambertini/2020	ESMO Open	Italy	Panel of experts or regional recommendations	Various	Practical suggestions on how to implement cancer care during the COVID-19 outbreak
Liu/2020	Indian J Surg	International	Panel of experts or regional recommendations	Various	An approach for the management of surgical patients in the context of the COVID-19 pandemic
Lou/2020	JCO Oncol Pract	US	Panel of experts or regional recommendations	Gastrointestinal	GI cancer treatment with the aim of minimizing patient risk during pandemic
Marijnen/2020	Radiother Oncol	International	Panel of experts or regional recommendations	Rectal	Radiotherapy treatment options for rectal cancer during the COVID-19 pandemic
Meattini/2020	ESMO Open	Italy	Panel of experts or regional recommendations	Various	Recommendations in order to keep cancer care as safe as possible for both patients and healthcare providers
Mohile/2020	Neuro Oncol	International	Panel of experts or regional recommendations	Glioma	To highlight opportunities to maximize the benefit and minimize the risk of glioma management during this pandemic and potentially, in the future
Monk/2020	Gynecol Oncol	US	Panel of experts or regional recommendations	Ovarian	Recommendation about alternative routes of therapy for ovarian cancer
Motlagh/2020	Arch Iran Med	Iran	Panel of experts or regional recommendations	Various	Two limit the exposure of cancer patients to medical environments, and modify the treatment modalities to reduce the probability of myelosuppression (delaying diagnostic and therapeutic services, shortening the treatment course, or prolonging the interval between treatment courses)
O’Cathail/2020	Clin Oncol	UK	Panel of experts or regional recommendations	Anorectal	Management of anorectal cancers provided by experts of UK
Patnaik/2020	Am J Hematology	International	Panel of experts or regional recommendations	Myelodisplastic/myeloproliferative syndrome	Recommendation about treatment by a panel of experts
Pino/2020	JCO Glob Oncol	Colombia	Panel of experts or regional recommendations	Various	Prioritization of adequate pathways for patients in low- and middle-income settings is critical
Pothuri/2020	Gynecol Oncol	US	Panel of experts or regional recommendations	Gynecologic	An expert panel convened to develop initial consensus guidelines regarding anti-neoplastic therapy during the COVID-19 pandemic with respect to gynecologic cancer care and clinical trials
Ramirez/2020	Int J Gynecol Cancer	International	Panel of experts or regional recommendations	Gynecologic	To share options in both the management and surveillance of patients diagnosed with gynecologic cancers during this time of global crisis
Sarkissian/2020	J Am Acad Dermatol	US	Panel of experts or regional recommendations	Dermatologic	Recommendations regarding dermatological surgery during COVID-19 pandemic
Shankar/2020	Asian Pac J Cancer Prev	International	Panel of experts or regional recommendations	Various	Specific precautions for cancer patients and cancer care providers to prevent spread
Soran/2020	Eur J Breast Health	US	Panel of experts or regional recommendations	Breast	A consensus and a statement that may guide breast care professionals (Magee-Breast Cancer Program)
Teoh/2020	World J Urol	EU	Panel of experts or regional recommendations	Bladder	Intravesical therapies recommendations
Ueda/2020	J Natl Compr Canc Netw	US	Panel of experts or regional recommendations	Various	The importance of organizational structure, preparation, agility, and a shared vision to provide cancer treatment to patients in the face of uncertainty and rapid change
Wang/2020	Crit Care	China	Panel of experts or regional recommendations	Various	Medical management strategies
Werner/2020	Otolaryngol Head Neck Surg	US	Panel of experts or regional recommendations	H&N	Care of cancer patients with head and neck cancers by US experts
Wu/2020	Otolaryngol Head Neck Surg	International	Panel of experts or regional recommendations	H&N	Point of view about head and neck cancer treatment during pandemic by Toronto and Wuhan hospitals
Akladios/2020	J Gynecol Obstet Hum Reprod	France	Multicenter cooperative groups	Gynecologic	Recommendations about curative treatment of cervical, ovarian and endometrial cancers according to stage and risk groups
Al-Shamsi/2020	Oncologist	International	Multicenter cooperative groups	Various	Consideration of risk and benefit for active intervention in the cancer population during an infectious disease pandemic must be individualized
Ardura/2020	Biol Blood Marrow Transplant	US	Multicenter cooperative groups	Hematologic cancers (stem cell transplantation)	Recommendations about hematopoietic stem cell transplantation during COVID-19
Glehen/2020	J Visc Surg	France	Multicenter cooperative groups	Peritoneal	RENAPE and BIG-RENAPE guidelines for peritoneal cancers
Penel/2020	Ann Oncol	France	Multicenter cooperative groups	Sarcoma	General recommendations for the management of sarcoma patients
Thureau/2020	J Bone Oncol	International	Multicenter cooperative groups	Bone metastasis	The COVID-19 crisis requires a reorganization of the health system, particularly in radiotherapy. A single 8Gy fraction is recommended for most clinical situations
Thoracic Surgery Outcomes Research Network/2020	Ann Thorac Surg	US	Multicenter cooperative groups	Thoracic	A document to offer guidance and to facilitate decisions when caring for patients with thoracic malignancies during the COVID-19 pandemic
Whisenant/2020	Cancer Cell	International	Multicenter cooperative groups	Thoracic Cancers	Evaluation of the impact of COVID-19 infection in patients with non-small cell lung cancer, small cell lung cancer, mesothelioma, thymic epithelial tumors, and thoracic carcinoid/neuroendocrine tumors. Thoracic Cancers International COVID-19 collaboration
Dingemans/2020	J Thorac Oncol	International	Multicenter cooperative groups	Lung	Multidisciplinary recommendations for the treatment of lung cancer during COVID-19 pandemic
Di Fiore/2020	Dig Liver Dis	France	Multicenter cooperative groups	Gastrointestinal tumors	Alternatives in the management of digestive cancers during COVID-19 pandemic. Clinical point of view of the French Intergroup
Hungria/2020	Hematol Transfus Cell Ther	Brazil	Multicenter cooperative groups	MM	Recommendations for the treatment of MM during COVID-19 pandemic. Recommendations from the ABHH Monoclonal Gammopathies Committee
Geskin/2020	J Am Acad Dermatol	USA	Multicenter cooperative group	Skin	Recommendations for the treatment of skin cancer patients during COVID-19 pandemic
Spolverato/2020	Surgery	Italy	Multicenter cooperative group	Surgical cancer patients	Management of surgical patients with cancer
De Azambuja/2020	ESMO Open	EU	Multicenter cooperative group	Breast	Recommendations for the treatment of breast cancer patients during COVID-19 pandemic
Catanese/2020	ESMO Open	EU	Multicenter cooperative group	Pancreas	Recommendations for the treatment of pancreatic cancer patients during COVID-19 pandemic
Jozaghi/2020	Head Neck	USA	Multicenter cooperative group	Endocrine	Recommendations for the treatment of endocrine surgical cancer patients during COVID-19 pandemic
Jereczek-Fossa/2020	Clin Oncol	Italy	Multicenter cooperative group	Various	Online questionnaires on how Lombardy radiotherapy departments have coped with COVID-19 pandemic
Van De Haar/2020	Nat Med	EU	Multicenter cooperative group	Various	A report on how seven comprehensive cancer centers in EU have organized their healthcare systems during COVID-19 pandemic
Al-Rashdan/2020	Adv Radiat Oncol	Canada	Multicenter cooperative group	Breast	Use of hypo-fractionation and accelerated partial breast irradiation for breast cancer during COVID-19 pandemic
Bartlett/2020	Ann Surg Oncol	US	Official guidelines or health authorities’ recommendations	Various	Considerations in management of cancer surgery cases during the COVID-19 pandemic
Bitar/2020	Future Oncol	Lebanon	Official guidelines or health authorities’ recommendations	Various	Recommendations for daily practice for the care of cancer patients relate to prevention of contamination, prioritization of patients, avoiding overcrowded clinics, ensuring the separation of oncology departments from other units, and management of palliative care patients
Coles/2020	Clin Oncol	International	Official guidelines or health authorities’ recommendations	Breast	Recommendations where RT is minimized and targeted to those with the highest risk of relevant breast recurrence, to protect our patients and health care professionals from potential exposure to COVID-19 as well as reducing the workload for health care providers and/or infrastructure
Kimmig/2020	J Gynecol Oncol	International	Official guidelines or health authorities’ recommendations	Gynecologic	Robot assisted surgery (RAS) may help to reduce hospital stay for patients that urgently need complex-oncological-surgery, thus making room for COVID-19 patients
Ribal/2020	Eur Urol	EU	Official guidelines or health authorities’ recommendations	Urologic	Position of EAU and reporting of guidelines recommendations during pandemic
Thomson/2020	Int J Radiat Oncol Biol Phys	International	Official guidelines or health authorities’ recommendations	H&N	This statement attempts to address the immediate impacts of the COVID-19 pandemic on HNC clinical practice. Practice recommendations for risk-adapted head and neck cancer radiotherapy
Troost/2020	Radiother Oncol	US + EU	Official guidelines or health authorities’ recommendations	Lung	ASTRO and ESTRO recommendations
Von Lillenfeld-Toal/2020	Leukemia	EU	Official guidelines or health authorities’ recommendations	Various	EHA Infectious Disease Scientific Working Group recommendations of cancer treatment
Yahalom/2020	Blood	International	Official guidelines or health authorities’ recommendations	Hematologic	Recommendations for alternative radiation treatment schemes: maintaining clinical efficacy and safety by increasing the dose per fraction while reducing the number of daily treatments
You/2020	Lancet Oncol	France	Official guidelines or health authorities’ recommendations	Various	In a situation where available care facilities are scarce, prioritization should involve the patients managed with curative-intent therapeutic strategies, and those with a life expectancy of 5 years or more, acknowledging that final decisions lie with the referring clinicians
Zaorsky/2020	Adv Radiat Oncol	US and UK	Official guidelines or health authorities’ recommendations	Prostate	A RADS framework (Remote visits, and Avoidance, Deferment, and Shortening of radiotherapy) was created and applied to determine the appropriate management for men with prostate cancer during the global COVID-19 pandemic
Chan/2020	Support Care Cancer	USA/Canada/Australia	Official guidelines or health authorities’ recommendations	Various	Three priority areas of survivorship care identified: triage of immediate needs of cancer survivors, tele-survivorship care, alternative models of care. A qualitative survey of Multinational Association of Supportive Care in Cancer (MASCC) Survivorship Study Group
Bergsland/2020	Pancreas	USA	Official guidelines or health authorities’ recommendations	Neuroendocrine tumors (NETs)/carcinomas (NECs)	Recommendations for the treatment of NETs/NECs during COVID-19 pandemic: Official guidelines of the North American Neuroendocrine Tumor Society
Terpos/2020	Leukemia	EU	Official guidelines or health authorities’ recommendations	Multiple Myeloma (MM)	Recommendations for the treatment of MM during COVID-19 pandemic: European Myeloma Network (EMN) Consensus Paper
Nguyen/2020	Cancers	International	Official guidelines or health authorities’ recommendations	Various	Practice proposal for the management of older cancer patients during COVID-19 pandemic. Proposal of the International Geriatric Radiotherapy Group
Desideri/2020	J Geriatr Oncol	International	Official guidelines or health authorities’ recommendations	Various	Recommendations for the treatment of older cancer patients during COVID-19 pandemic. Global perspective of the Young International Society of Geriatric Oncology (SIOG)
Vecchione/2020	ESMO Open	EU	Official guidelines or health authorities’ recommendations	Colorectal	ESMO recommendations: redefinition of diagnostic and therapeutic algorithms in colorectal cancer

H&N, head and neck; US, United States.
